# Multiple pygmy blue whale acoustic populations in the Indian Ocean: whale song identifies a possible new population

**DOI:** 10.1038/s41598-021-88062-5

**Published:** 2021-04-22

**Authors:** Emmanuelle C. Leroy, Jean-Yves Royer, Abigail Alling, Ben Maslen, Tracey L. Rogers

**Affiliations:** 1 Evolution and Ecology Research Centre, School of Biological, Earth and Environmental Sciences, University of New South Wales, Sydney, Australia; 2 University of Brest and CNRS Laboratoire Geosciences Ocean, IUEM, 29280 Plouzané, France; 3 Biosphere Foundation, P.O. Box 112636, Campbell, CA 95011-2636 USA; 4 Mark Wainwright Analytical Centre, University of New South Wales, Sydney, Australia

**Keywords:** Animal migration, Biogeography, Marine mammals, Marine biology

## Abstract

Blue whales were brought to the edge of extinction by commercial whaling in the twentieth century and their recovery rate in the Southern Hemisphere has been slow; they remain endangered. Blue whales, although the largest animals on Earth, are difficult to study in the Southern Hemisphere, thus their population structure, distribution and migration remain poorly known. Fortunately, blue whales produce powerful and stereotyped songs, which prove an effective clue for monitoring their different ‘acoustic populations.’ The DGD-Chagos song has been previously reported in the central Indian Ocean. A comparison of this song with the pygmy blue and Omura’s whale songs shows that the Chagos song are likely produced by a distinct previously unknown pygmy blue whale population. These songs are a large part of the underwater soundscape in the tropical Indian Ocean and have been so for nearly two decades. Seasonal differences in song detections among our six recording sites suggest that the Chagos whales migrate from the eastern to western central Indian Ocean, around the Chagos Archipelago, then further east, up to the north of Western Australia, and possibly further north, as far as Sri Lanka. The Indian Ocean holds a greater diversity of blue whale populations than thought previously.

## Introduction

Commercial whaling in the twentieth century brought blue whales (*Balaenoptera musculus*) to the brink of extinction; for instance, in the Southern Hemisphere it is estimated that less than 0.15% of the blue whale population survived whaling^1^. Despite increases in blue whale populations at a global scale, their recovery remains slow and they are classified as Endangered by the IUCN Red List^2^. Despite their enormous size, blue whales have been difficult to observe in the Southern Hemisphere; thus, for some regions, their population structure, distribution and migration routes remain poorly understood. In particular, little is known about the blue whales in the northern Indian Ocean^3^.

To overcome the limitations of classical visual surveys, passive acoustic monitoring proves an efficient method to monitor this vocal species^4^. Blue whales produce powerful and stereotyped songs, that they repeat in sequences for hours to days. Each blue whale population has a distinct vocal signature, which can be used to distinguish and monitor different ‘acoustic populations’ or ‘acoustic groups’^5^. The mechanisms that have led to the geographic variation in their song types is unknown (*e.g.,* physical and environmental adaptation, and/or cultural transmission). Regardless of whether song variation is a driving force, or a consequence of reproductive isolation or similar events, understanding song variation across the species’ range can provide valuable insight to conservation management of the species.

The Indian Ocean has an incredible diversity of blue whale acoustic populations^6–11^. Until very recently, there were four recognized blue whale populations from two subspecies: the Antarctic blue whale (*B. m. intermedia*), that is believed to produce the same song across the Southern Hemisphere; and three acoustic populations of the pygmy blue whale (*B. m. brevicauda*). The pygmy blue whale populations are distinguishable only acoustically; they do not display morphological differences and genetic data are sparse^12^. One population dwells in the southwestern Indian Ocean (SWIO) and is characterized by the Madagascan or type-9 song. A second population dwells in the southeastern Indian Ocean (SEIO) and is characterized by the Australian or type-8 song. Finally, a third population, and possibly a separate subspecies (*B. m. indica*), dwells in the northern and central Indian Ocean (NIO), and is characterized by the Sri Lankan or type-7 song^5^. Very recently, evidence for a fourth pygmy blue whale acoustic population were found in the northwestern Indian Ocean (NWIO) in the Arabian Sea off Oman, in the southwestern Indian Ocean off Madagascar, as well as in the central Indian Ocean on the west side of the Chagos Archipelago (DGN site, see below)^13^. Together with the Antarctic blue whales, all five of these blue whale populations are sympatric in the central Indian Ocean^8,11,13^.

A possible sixth blue whale song, the ‘Diego Garcia Downsweep’ (DGD) referred to here as Chagos song, has been recorded in the central Indian Ocean, off Diego Garcia, an atoll in the Chagos Archipelago^14^. The Chagos song was initially considered to be a variant of the Madagascan pygmy blue whale song^5^. Sousa and Harris (2015) re-examined the song, and compared its temporal and spectral properties with the vocalizations of other baleen whale species known to dwell in the area (*i.e.*, the Bryde’s whales (*Balaenoptera edeni*), humpback whales (*Megaptera novaengliae*), minke whales (*B. acutorostrata*), sei whales (*B. borealis*), fin whales (*B. physalus*) and blue whales (*B. musculus*)). They strongly suggested that the Chagos song was a new blue whale song and not a variant of the Madagascan pygmy blue whale song (see^14^ for detailed comparison).

Sousa and Harris (2015) however, described a second type of vocalization off Diego Garcia, the ‘Diego Garcia Croak’ (DGC)^14^. Where the Chagos song is a three-unit song, the DGC song is typically a single-unit song. The first part of the Chagos song shares acoustic features with the DGC song. They are similar in duration (approximately 4 s), frequency range (approximately 15 to 50 Hz)^14^, and are both described as amplitude-modulated in structure^14–17^. Thus, it is possible that these songs are produced by the same whale species. As the DGC song has been recently attributed to the Omura’s whale (*B. omurai*) based on acoustic similarity with the Omura’s whales recorded off Madagascar^15,17^, one could argue that the Chagos song is an Omura’s and not a blue whale song.

**Figure 1:**
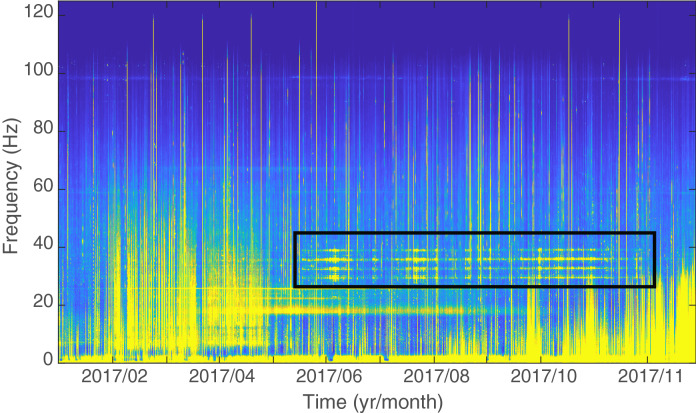
Long-term spectrogram of data recorded on the eastern side of the Chagos Archipelago (DGS site) in 2017: Chagos songs are a major component of the central Indian Ocean underwater soundscape and have been so for nearly two decades. The acoustic energy in the parallel horizontal frequency bands outlined in the black box are Chagos songs. Spectrogram parameters: 6 h averaging window, 50% overlap, FFT window of 120 s.

In the event that the Chagos song is produced by the blue whale, and given its huge contribution to the underwater soundscape around the Chagos Archipelago (Fig. 1), it suggests that there is a previously unknown (pygmy) blue whale population in the central Indian Ocean. If this was the case, we hypothesize that the Chagos song should: (1) possess acoustic characteristics more like the blue whale songs than the Omura’s whale songs; and (2) that the presence of Chagos songs in the Indian Ocean soundscape would be consistent with the behaviour of other blue whale populations. For example: (2a) the Chagos songs should be detected across a relatively wide spatial distribution, reflecting the wide-ranging habitat of the blue whale; (2b) the song occurrence should show seasonal variation within the year; and (2c) the seasonality in song occurrence should remain relatively stable across years.

**Figure 2:**
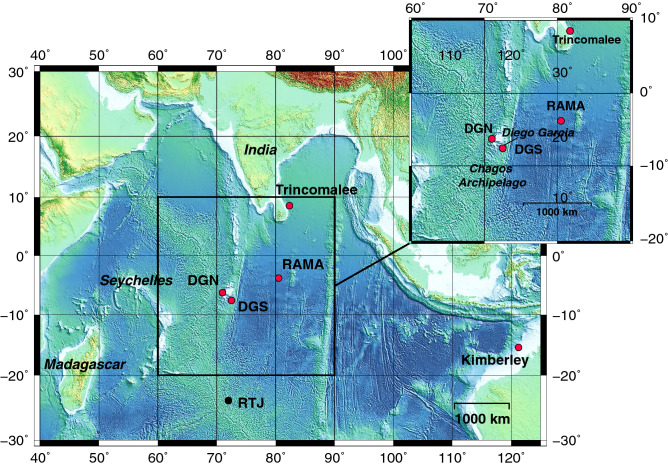
Study area and locations of the hydroacoustic stations (circles) used in this study to explore the presence of Chagos songs: in the equatorial waters, off Trincomalee, Sri Lanka; in the tropical waters: RAMA and the western (DGN) and eastern (DGS) sides of Chagos Archipelago; Kimberley, at the north of Western Australia, at the limit between the tropical and subtropical waters; and RTJ in the subtropical waters of the central Indian Ocean. Red circles indicate the sites where Chagos songs have been recorded.

To explore this question, we examine the acoustic structure, frequency and temporal characteristics of the Chagos song, and compare it to the pygmy blue whale song-types of the Indian Ocean (Madagascan, Sri Lankan and Australian song-types), and to Omura’s whale song-types recorded in the tropical waters of the South Atlantic Ocean^18^, off Ascension Island; off Madagascar^15^; around the Chagos Archipelago at Diego Garcia sites^14^; and off Kimberley, Western Australia^16^. Then we investigate the presence of the Chagos songs at several locations in the Indian Ocean: (1) one site in the Equatorial waters, off Trincomalee, Sri Lanka; (2) three sites in the tropical waters: at RAMA (03.5$^{\circ }$ S, 080.3$^{\circ }$ E, 16 months of continuous hydroacoustic data) and two sites further south-west, off the Chagos Archipelago (DGN, 06.3$^{\circ }$ S, 071.0$^{\circ }$ E and DGS, 07.6$^{\circ }$ S, 072.5$^{\circ }$ E, up to 17 years of continuous data); (3) one site further south-east, at the limit between the tropical and subtropical waters, off Kimberley, Western Australia (15.5$^{\circ }$ S, 121.25$^{\circ }$ E); and (4) one site further south, in the subtropics at RTJ (24.0$^{\circ }$ S, 072.0$^{\circ }$ E, one year of data); Fig. 2).

## Results

### Song description: terminology

#### Song organisation

Blue whale vocal sequences are traditionally referred to as ‘calls’^19–21^, however, as they meet the criterion of ‘song’ as used in the bioacoustic community^22^, in this study we use the term ‘song’ to refer to regularly-repeated whale vocalisations. The song is repeated in a sequence with regular intervals, defined as the Inter-Call Interval (ICI), measured as the time interval between the beginning of the one song and the beginning of the following song. Note that although we use the term ‘song’, we chose to keep the definition ‘ICI’ as this nomenclature is used traditionally in the whale literature, rather than ‘ISI’, which usually designates Inter-Series (or Sequence) Interval. Songs are composed of units and we used the term ‘unit’ to designate parts of the song that are separated by a silence (see reviewed criteria in^23^). Units were divided into subunits: subunits are defined as such when there is a sudden change in the sound structure for instance becoming harmonic or noisy.

#### Sound types

A sound can be of different types: (1) the simpler one is the simple tone, which is either pure, with the same frequency all along, or showing frequency and/or amplitude modulations; (2) harmonic sounds are sounds with multiple tones at frequencies that are integer multiples of the frequency of the original wave, called the fundamental frequency ($F_{0}$). When one of the harmonics has a greater amplitude than the others, it is called ‘resonance frequency’; (3) pulsed sounds are, as defined in^24^, the repetition of similar “pulses” or short signals with a constant pulse rate, often aurally perceived by humans as amplitude modulated sounds. On spectrogram representation, using a long analysis time window, these sounds are characterized by sidebands with regular spacing. The frequency difference ($\Delta f$) between each sideband is the pulse rate of the sound. In their recent study, Patris et al. made the difference between what they defined as ‘tonal pulsed sounds’ and ‘non-tonal pulsed sounds’^24^. Following their criterium, the sidebands of the tonal pulsed sounds show a harmonic relationship, meaning that the frequency of each sideband divided by the pulsed rate is a positive integer. If it is not the case, then the sound is a non-tonal pulsed sound.

#### Nonlinear phenomena

Nonlinear phenomena are observed in a variety of birds^25^, anurans^26^ and mammals^27,28^, including marine mammals (*e.g.*, manatee^29^) and more particularly cetaceans (right whales^30,31^, killer whales^30,32^ and humpback whales^33^). They have been well described by a variety of authors^27,34^ and include: (1) frequency jumps, that are characterized by sudden $F_{0}$ changes which moves up or down abruptly and discontinuously, and is different from continuous, smooth modulation^27^; (2) subharmonics, that are additional spectral components and can suddenly appear at integer fractional values of an identifiable $F_{0}$ (*e.g.*, $F_{0}/2$, $F_{0}/3, \ldots$) and as harmonics of these values. On a spectrogram, it results as bands of energy evenly spaced below $F_{0}$ and between its harmonics throughout the spectrum; (3) biphonation, that is the simultaneous occurrence of two independent fundamental frequencies $F_{0}$ and $G_{0}$. Biphonation can be visible on a spectrogram as two distinct frequency contours^35^. Alternatively, if one source ($F_{0}$) vibrates at a much lower frequency than the other ($G_{0}$), biphonation will appear as visible sidebands at linear combinations of $F_{0}$ and $G_{0}$ (m$G_{0}$ ± n$F_{0}$, where m and n are integers), because the airflow is then modulated by the frequency difference. This is equivalent to considering that the lower $F_{0}$ amplitude-modulates the higher frequency $G_{0}$ (carrier frequency)^28^; (4) finally, deterministic chaos are broadband, noise-like segments. These episodes of non-random noise appear via abrupt transitions and can also contain some periodic energy, which appears as banding in a spectrogram. In extreme cases there are no repeating periods at all^27,34^.

### Analysis of the Chagos song and comparison with the Indian Ocean pygmy blue whale song types and Omura’s whale song types

#### Chagos song

**Figure 3:**
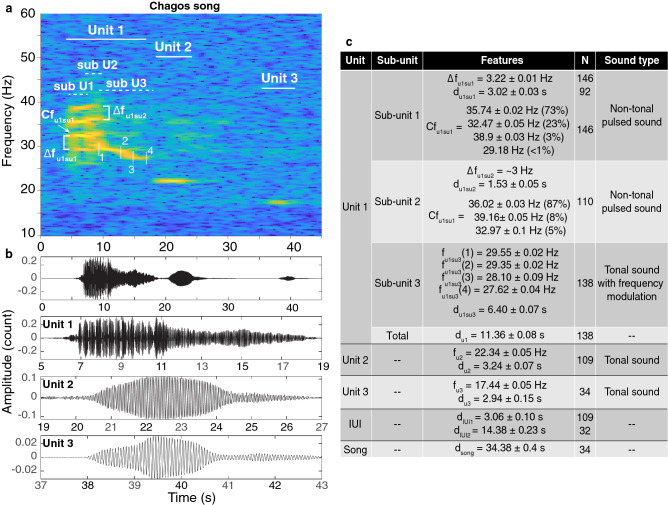
Spectrogram (**a**) and waveforms (**b**) of a Chagos song recorded on the eastern side of the Chagos Archipelago (DGS) in August 2017. Detailed waveforms show the signal structure of the units within the song. Spectrogram parameters: Hamming window, 1024-point FFT length, 90% overlap. Note that the axes differ among plots. (**c**) Measurements (mean ± standard error (s.e.)) of the acoustic features. *N* is the number of measurements, $u_{i}su_{j}$ stands for $unit_{i} subunit_{j}$ where *i* and *j* are the unit and subunit numbers, $\Delta f$ designates the frequency difference between the sidebands, *f* and *d* are the frequency and duration of the feature indicated in subscript, and when present, the number in brackets refers to the point measured as indicated on the spectrogram. $F_{x}$ or $G_{x}$ designate the *x*th harmonic of a sound, and *Cf* designates the carrier frequency of a sound.

The Chagos song was composed of 3 units (Fig. 3). The 3-unit song was repeated in stereotyped series with an ICI of $190.79 \pm 1.49$ s (Fig. 7b).

The first unit of the Chagos song is divided into 3 subunits (Fig. 3): in 2017, subunit 1 was pulsed with a rate $\Delta f_{u1su1}$ = 3.22 ± 0.01 Hz. Using Patris *et al.* ’s criterion^24^, we concluded that this subunit is a non-tonal pulsed sound, since the sidebands do not have a harmonic relationship. The carrier frequency (where the peak of energy lies) was 35.74 ± 0.02 Hz for 73% of the measured songs, 32.47 ± 0.05 Hz for 23% of the songs and 38.9 ± 0.06 Hz for 3% of the measured songs. One song had a carrier frequency of 29.18 Hz. This subunit 1 lasted 3.02 ± 0.03 s in duration. Subunit 2 was often less obvious (likely due to propagation effects, lower source level or possibly to deterministic chaos) so that it could not be measured for all of the songs sampled; it is also a short (1.53 ± 0.05 s) non-tonal pulsed unit with a pulse rate ($\Delta f_{u1su2}$) of approximately 3 Hz and a slightly different carrier frequency, induced by a frequency jump. The carrier frequency was of 36.02 ± 0.03 Hz for 87% of the measurements, 39.16 ± 0.05 Hz for 8% of the measured songs and 32.97 ± 0.1 Hz for 5%. Finally, subunit 3 was a tonal unit showing a frequency modulation. The subunit started at 29.55 ± 0.02 Hz down to 29.35 ± 0.02 Hz over approximately 3.5 s, then down to 28.10 ± 0.09 Hz as a decrease to 27.62 ± 0.04 Hz over 3 s. The total duration of this subunit was 6.40 ± 0.07 s, and the total duration of the unit 1 was 11.36 ± 0.08 s.

Unit 2 was a pure tone following after a silence of 3.06 ± 0.1 s. Its peak frequency was 22.34 ± 0.05 Hz and its duration was 3.24 ± 0.07 s. Finally, unit 3, also a pure tone, followed after a silence of 14.38 ± 0.23 s. It had a peak frequency of 17.44 ± 0.05 Hz and lasted 2.94 ± 0.15 s. The third unit was sometimes absent. This could be due to a variation in the song or due to propagation losses. When unit 3 was present, the total song duration was 34.38 ± 0.4 s.

**Figure 4:**
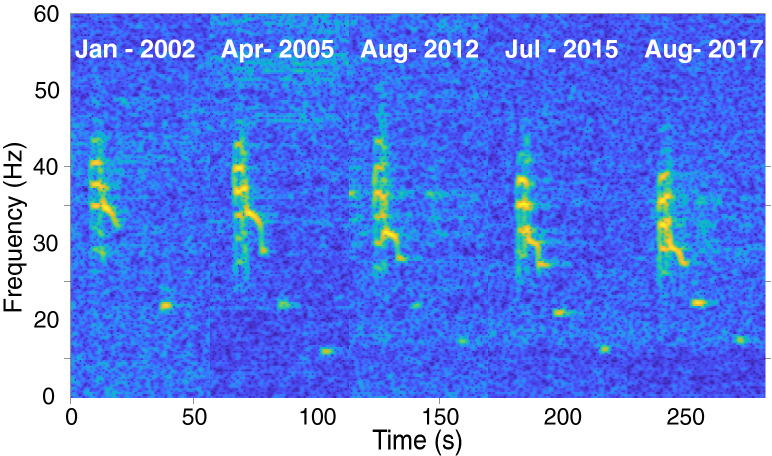
The decline in frequency of the Chagos song from 2002 to 2017: spectrogram representation of five songs recorded at Diego Garcia in years 2002, 2005, 2012, 2015 and 2017. Spectrogram parameters: Hamming window, 1024-point FFT length, 90% overlap.

The frequency for the beginning of the third subunit of the unit 1 of the Chagos song (point 1 in Fig. 3a) decreased by approximatively 0.33 Hz/year across years (Fig. 4).This phenomenon will be examined in details in a further study.

#### Indian Ocean pygmy blue whale songs

This section describes the structural, temporal and frequency features of the pygmy blue whale song-types commonly reported in the Indian Ocean. Note that as the frequency of at least parts of these songs are known to vary within and across years^36–41^, the frequency values obtained here are only valid for the years sampled.

**Figure 5:**
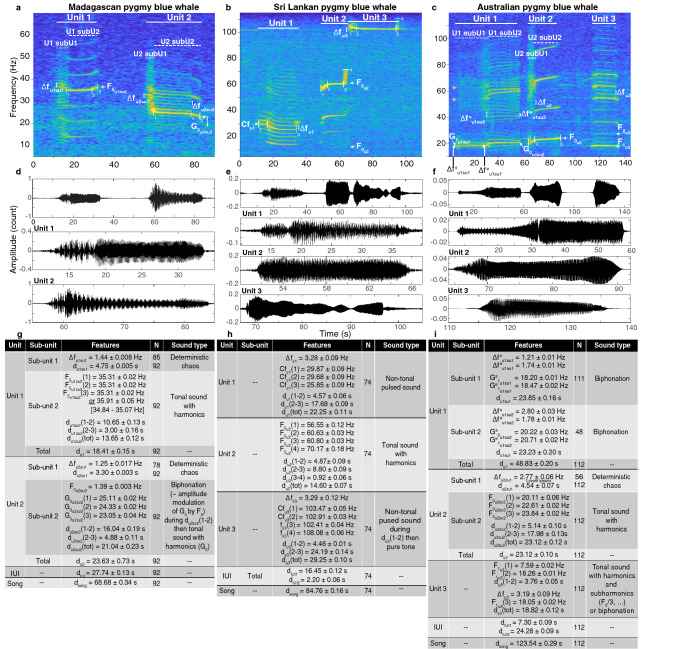
Spectrograms (upper panels) and waveforms (middle panels) of the song of the Madagascan, Sri Lankan and Australian pygmy blue whales, including detailed waveforms to show the internal signal structure. The Madagascan song was recorded off Crozet Island (CTBTO records, site H04S1) in April 2004, the Sri Lankan song was recorded at DGN (CTBTO records, site H08N1) in April 2009 and the Australian song was recorded at Perth Canyon in March 2008 (IMOS records). (Spectrogram parameters: Hamming window, 1024-point FFT length, 90% overlap. Note that the axes differ among plots.) And measurements (mean ± s.e., lower panels) of the acoustic features of the different song types. *N* is the number of measurements, $u_{i}su_{j}$ stands for $unit_{i} subunit_{j}$ where *i* and *j* are the unit and subunit numbers, $\Delta f$ designates the frequency difference between the sidebands, *f* and *d* are the frequency and duration of the feature indicated in subscript, and when present, the number in brackets refers to the point measured as indicated on the corresponding spectrogram. $F_{x}$ or $G_{x}$ designate the *x*th harmonic of a sound, and *Cf* designates the carrier frequency of a sound.

**Madagascan pygmy blue whale** The Madagascan pygmy blue whale song had 2 units (Fig. 5a). Unit 1 was divided into 2 subunits. In 2004, subunit 1 was a noisy pulsed sound, characteristic of deterministic chaos, with a pulse rate $\Delta f_ {u1su1}$ = 1.44 ± 0.01 Hz and of 4.76 ± 0.005 s duration. Subunit 2 was a tonal sound with harmonics. Its $F_ {0}$, estimated as the mean frequency difference between the harmonics, was 7.04 ± 0.005 Hz. The maximum energy was in the $F_ {5}$ (resonance frequency), which commenced at 35.31 ± 0.02 Hz and remained stable over 10.65 ± 0.13 s ($F_{5_{u1su2}}$ in Fig. 5a). The frequency then remained stable over another 3.00 ± 0.16 s or in some songs increased to 35.91 ± 0.05 Hz [range = 34.84–37.05 Hz]. The total duration of subunit 2 was 13.65 ± 0.12 s, and unit 1 was 18.41 ± 0.15 s.

Unit 2 followed after 27.74 ± 0.13 s. It had 2 subunits. Subunit 1 was a noisy pulsed sound, identified as deterministic chaos, it had a pulsed rate of $\Delta f_ {u1su1}$ = 1.25 ± 0.017 Hz, and a duration of 3.30 ± 0.05 s. Subunit 2 was a complex harmonic-like signal, with sidebands spaced by $\Delta f_ {u2su2}$ = 1.39 ± 0.003 Hz. Calculations of the ratio of the sideband frequencies over $\Delta f$ show that these 1.39 Hz-spaced bands do not have a harmonic relationship. However, relatively higher energy lies in frequency bands that have a harmonic relationship, where the band with the greatest energy started at 25.11 ± 0.02 Hz and ended at 24.33 ± 0.02 Hz ($G_{3_{u2su2}}$ on Fig. 5). On the low signal-to-noise ratio (SNR) songs, only the harmonic bands were visible, this explains why this unit has been described previously as a harmonic signal when it is not^7^. The complex structure of subunit 2 can be explained by a phenomenon of biphonation, where there are two concurrent frequencies, with a lower fundamental frequency ($F_{0}$) of 1.39 Hz, a higher fundamental frequency ($G_{0}$) of 8.37 Hz (resonance frequency $G_{3}$ starting at 25.11 Hz), and the sidebands at m$G_{0}$ ± n$F_{0}$ consistent with the amplitude modulation of $G_{0}$ by $F_{0}$. This biphonation event lasted for 16.04 ± 0.19 s. Finally, subunit 2 ended in a tonal sound with the harmonics ($G_{0}$ = 7.94 Hz ± 0.003 Hz), that decreased in frequency from 24.30 ± 0.02 Hz to 23.05 ± 0.04 Hz over 4.88 ± 0.11 s (measured for the harmonic where there is the greatest energy ($G_{3_{u2su2}}$)). Unit 2 was 23.63 ± 0.73 s in duration. The total duration of the Madagascan pygmy blue whale song was 68.68 ± 0.34 s.

**Sri Lankan pygmy blue whale** The Sri Lankan pygmy blue whale song had 3 units (Fig. 5b). In 2009, unit 1 was a pulsed, non-tonal sound of a duration of 22.25 ± 0.11 s. The pulse rate was $\Delta f_{u1}$ = 3.28 ± 0.09 Hz. The carrier frequency of unit 1 started at 29.87 ± 0.09 Hz (‘Cf’ on Fig. 5b), and slightly down swept to 29.68 ± 0.09 Hz over 4.57 ± 0.06 s, then the frequency decreased to 25.85 ± 0.09 Hz over 17.68 ± 0.09 s.

Unit 2 followed after 16.45 ± 0.12 s of silence. Unit 2 was a tonal sound with harmonics spaced by 12.21 ± 0.08 Hz. The maximum of energy was in the $F_{5_{u2}}$ and started at 56.55 ± 0.12 Hz, increased to 60.63 ± 0.03 Hz over 4.87 ± 0.09 s, then increased to 60.80 ± 0.03 Hz over 8.80 ±0.09 s, and finally increased sharply to 70.13 ± 0.18 Hz overe 0.92 ± 0.06 s. Unit 2 was 14.60 ± 0.07 s in duration.

Unit 3 followed after 2.20 ± 0.06 s of silence. It started as a non-tonal pulsed sound lasting 4.46 ± 0.01 s, with a pulse rate $\Delta f_{u3}$ = 3.29 ± 0.12 Hz and a carrier frequency starting at 103.47 ± 0.05 Hz and slightly decreasing to 102.91 ± 0.03 Hz. It then continued as a pure tone starting at 102.63 ± 0.05 Hz down to 102.41 ± 0.04 Hz during 24.19 ± 0.14 s and then suddenly peaked to 108.08 ± 0.06 Hz. Unit 3 lasted 29.25 ± 0.10 s in total, and the entire song was 84.76 ± 0.16 s in duration.

**Australian pygmy blue whale** The Australian pygmy blue whale song is the most complex of the pygmy blue whale songs. It is traditionally described as a 3-unit signal, although multiple variations in the unit order (or syntax) are found^42^. The song variants change the order and repetition of the unit types. Here, for simplicity, we selected and thus described only the common traditional 3-unit song (Fig. 5c).

Unit 1 was 48.83 ± 0.20 s in duration. It had 2 subunits: subunit 1 was a pulsed sound, with a pulse rate $\Delta f^{s}_{u1su1}$ = 1.21 ± 0.01 Hz at the beginning of the subunit, pulsing accelerated to reach $\Delta f^{e}_{u1su1}$ = 1.71 ± 0.01 Hz at the end of the unit. Following the ratio “band frequency/pulse rate” criterion, this unit is a non-tonal pulsed sound. However, it is a biphonation sound, as higher energy bands, which do have a harmonic relationship and are spaced by approximately 9 Hz, are obvious on the spectrogram (grey arrows on Fig. 5). The higher fundamental frequency $G_{0}$ was at $\sim$ 9.10 Hz. The resonance frequency of this harmonic sound was the $G_{1_{u1su1}}$. It started at 18.20 ± 0.02 Hz and ended at 18.47 ± 0.02  Hz, and was 23.85 ± 0.16 s in duration. Subunit 2 is also a biphonation sound, with a $F_{0}$ at 2.80 ± 0.03 Hz at the beginning of the unit ($\Delta f^{s}_{u1su2}$ in Fig. 5c), decreasing to 1.78 ± 0.01 Hz at the end of the subunit ($\Delta f^{e}_{u1su2}$), which gives an impression of a decreasing pulse rate when listening to the song. This change in $F_{0}$ frequency creates the complicated pattern of intersecting sidebands toward the end of unit 2. The harmonic bands are spaced by approximately 20 Hz (= $G_{0}$, precise measurements are given below). Subunit 2 had two variations: subunit 2 was continuous in 42.9% of the sampled songs, but was interrupted by a short gap in 57.1%. In the continuous subunit case (N = 48), the fundamental frequency ($G_{0_{u1su2}}$), which is here the band with the most energy, started at 20.22 ± 0.03 Hz and ended at 20.71 ± 0.02 Hz. The subunit lasted 23.26 ± 0.2 s. In the interrupted subunit case (N = 64), the fundamental frequency ($G_{0_{u1su2}}$) started at 20.12 ± 0.03 Hz and slightly increased to 20.44 ± 0.02 Hz over 15.27 ± 0.21 s. Then, there was a silence of 3.32 ± 0.08 s followed by the resumption of the subunit at 20.29 ± 0.03 Hz increasing to 20.48 ± 0.17 Hz over 5.71 ± 0.17 s. In this case, the total duration of the subunit (gap included) was 24.31 ± 0.14 s.

Unit 2 followed after 7.30 ± 0.09 s. It started as a slightly noisy pulsed sound (possibly deterministic chaos) with a rate $\Delta f_{u2}$ = 2.77 ± 0.06 Hz during 4.54 ± 0.07 s, then continued as a tonal sound with harmonics. The $F_{0_{u2}}$ started at 20.11 ± 0.06 Hz, increased to 22.61 ± 0.02 Hz over 5.14 ± 0.10 s, and then slowly increased to 23.84 ± 0.02 Hz over 23.84 ± 0.02 s. Unit 2 was 23.12 ± 0.12 s in duration.

Unit 3 followed after 24.28 ± 0.09 s of silence. It started as a tonal sound with harmonics spaced by 8.93 ± 0.05 Hz. The resonance frequency ($F_{1_{u3}}$) started at 7.59 ± 0.02 Hz then increased to 18.26 ± 0.01 Hz over 3.76 ± 0.05 s, with the appearance of sidebands with non-harmonic relationship, spaced by $\Delta f_{u3}$ = 3.19 ± 0.09 Hz. These non-tonal pulses stopped approximately 3.5 s before the end of the unit, which ends on the harmonic sound, slightly down swept to 18.05 ± 0.02 Hz. These sidebands could be subharmonics, ($F_{0}/3, 2F_{0}/3$, etc). Alternatively, they could suggest a biphonation sound. This third unit lasted 18.82 ± 0.12 s in duration, and the whole 3-unit song was 123.54 ± 0.29 s in duration.

#### Omura’s whale songs

**Figure 6:**
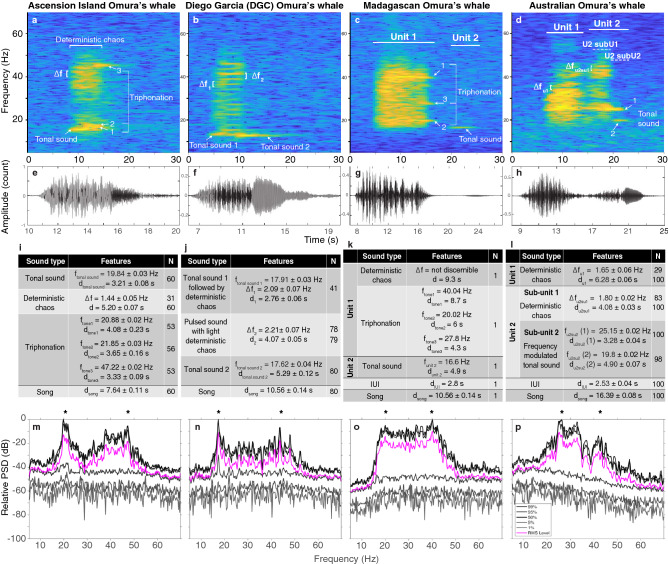
Spectrograms (**a**–**d**), waveforms (**e**–**h**), acoustic measurements (mean ± (s.e.)—**i**–**l**), and Power Spectral Density (PSD—**m**–**p**) of the songs of the Omura’s whales from Ascension Island, Madagascar, Diego Garcia and Australia. The stars on the PSD (**m**–**p**) outline the peaks of energy. The Ascension Island song was recorded off Ascension Island (CTBTO records, site H10N1) in November 2005, the Madagascar song was recorded off Madagascar in December 2015 and provided by S. Cerchio, the Diego Garcia DGC song was recorded at DGN (CTBTO records, site H08N1) in October 2003 and the Australian song was recorded at Kimberley site in March 2013 (IMOS records). For the panels (**a**–**d**) and (**i**–**j**): *N* is the number of measurements, $u_{i}su_{j}$ stands for $unit_{i} subunit_{j}$ where *i* and *j* are the unit and subunit numbers, $\Delta f$ designates the frequency difference between the sidebands, *f* and *d* are the frequency and duration of the feature indicated in subscript, and when present, the number in brackets refers to the point measured as indicated on the corresponding spectrogram. (Spectrogram parameters: Hamming window, 1024-point FFT length, 90% overlap. Note that the axes differ among plots).

All Omura’s whale songs showed energy between 15 and 55 Hz and peaks of energy around 20 and 40–45 Hz (Fig. 6 lower panels).

**Ascension Island Omura’s whale** Omura’s whale songs recorded in 2005 off Ascension Island started as a tonal sound at 19.84 ± 0.03 Hz. This tone was 3.21 ± 0.08 s in duration but less than 1 s after its beginning, it was overlapped by a noisy pulsed sound, typical of deterministic chaos. The pulse rate was estimated at $\Delta f$ = 1.44 ± 0.05 Hz. This deterministic chaos lasted for 5.20 ± 0.07 s. Finally, 2.65 ± 0.06 s after the beginning of the song, three tonal components appeared at harmonically independent frequencies, characteristic of triphonation: two tones starting simultaneously, one at 20.88 ± 0.02 Hz and the other at 21.85 ± 0.03 Hz, lasting respectively 4.08 ± 0.23  s and 3.65 ± 0.16 s, and a third tone starting a bit later, 4.48 ± 0.07 s after the beginning of the song, at a frequency of 47.22 ± 0.03 Hz and lasting 3.33 ± 0.09 s. The duration of the total component was 7.64 ± 0.11 s (Fig. 6a).

**Madagascan Omura’s whale song** The following description of the Madagascan Omura’s whale song uses the description provided by Moreira et al.^18^ and observation from the spectrogram (Fig. 6b). In 2015, Cerchio et al. described the Madagascan Omura’s whale song recorded in 2013–2014 as a single-unit amplitude-modulated low frequency vocalization, with a 15–50 Hz bandwidth^15^. More recently, Moreira et al. reported a 2-unit song, with the first unit commencing as an amplitude-modulated component with bimodal energy at 20.75 Hz and 40.04 Hz, followed by a harmonic component with a low harmonic at 20.0 Hz and an upper harmonic at 41.0 Hz, as well as an additional tone at $\sim$ 30 Hz. Unit 1 was characterized as sometimes followed by a tonal unit at 16 Hz^18^. The ICI was 189.7 s (s.d. 16.47 s, measured from 118 series with $\ge$ 20 consecutive songs) and ranged from 145.5 to 237.6 s^43^.

Based on the song example recorded in December 2015 in Nosy Be, Madagascar, and provided by S. Cerchio, we observed a 2-unit song (Fig. 6b). The first unit started as chaotic, with no visible sidebands. After $\sim$ 3 s the signal had a bi- or triphonation event (whilst the deterministic chaos still continues), with first a tone at 40.04 Hz, another tone with a harmonic relationship at 20.02 Hz but starting circa 2.6 s later and a third one at 27.8 Hz starting 4.4 s after the beginning of the first tone, whilst the chaotic sound ends (the chaotic sound lasted circa 9.3 s). The tones of the bi- or triphonic sound all ended at the same time, 11.7 s after the beginning of the song. The second unit seems to be optional^15,17,18,44^. It followed after 2.8 s silence. It was a tonal sound of 4.9 s in duration with a peak frequency of 16.6 Hz. (Note that the observations here are purely qualitative since only based on 1 song).

**Diego Garcia Omura’s whale song (DGC)** The ‘Diego Garcia Croak’—DGC—recently attributed to the Omura’s whales^17^ was comprised of one unit (Fig. 6c), although sometimes a second unit was present. The first unit was tonal at the start, with a frequency of 17.91 ± 0.03 Hz, quickly becoming a noisy pulsed sound, characteristic of deterministic chaos, with a pulsed rate of 2.09 ± 0.07 Hz estimated on 41 songs. This chaotic component was 2.76 ± 0.06 s in duration to then became pulsed, although still slightly noisy, with a pulse rate of 2.21 ± 0.005 Hz. This part showed a peak of energy around 19.46 ± 0.08 Hz, and another one around 43.51 ± 0.11 Hz (Fig. 6c, lower panel), and lasted 4.07 ± 0.05 s. Finally, the unit ended as a tonal sound at 17.62 ± 0.04 Hz lasting 5.29 ± 0.12 s. This whole unit had a duration of 10.56 ± 0.14 s. In some occurrences (N = 12), a second tonal unit was present after a silence of 39.89 ± 0.5 s. Unit 2 started at 13.51 ± 0.06 to 13.46 ± 0.04 Hz and lasted for 3.81 ± 0.20 s. When the second unit was present, the entire song was 54.74 ± 0.19 s in duration. Note that in our study, out of the 80 songs measured only 12 had unit 2.

**Australian Omura’s whale song** The Omura’s whale song recorded in 2013 off western Australia had two units (Fig. 6d). Unit 1 was a noisy pulsed sound with a pulse rate of 1.65 ± 0.06 Hz with deterministic chaos, and a duration of 6.28 ± 0.06 s.The peak in energy was at 25.32 ± 0.14 Hz followed by a gap of 2.53 ± 0.04 s, and then a second noisy pulsed unit, with a pulsed rate of 1.80 ± 0.02 Hz estimated on 83 songs. This unit lasted 4.08 ± 0.03 s and had a peak of energy at 25.25 ± 0.18 Hz and another one at 41.20 ± 0.18 Hz (Fig. 6d, lower panel). During the last third of unit 2, the song transitioned to a tonal sound, starting at 25.15 ± 0.02 Hz and swept down to 25.07 ± 0.02 Hz over 3.28 ± 0.04 s, then abruptly decreased to 19.8 ± 0.02 Hz and became tonal for 4.90 ± 0.07 s, forming a z-shape on the spectrogram representation. The whole song was 16.39 ± 0.08 s in duration.

#### Deterministic chaos

We classified deterministic chaos as: ‘slight’, where sidebands were easily distinguished but the sound was noisy; ‘moderate’, where the sidebands were visible but difficult to measure; and ‘strong’, where the sound had no discernible structure. Where deterministic chaos was present, we identified its persistence, defined as the proportion of deterministic chaos over the duration of a song^31^.

**Figure 7:**
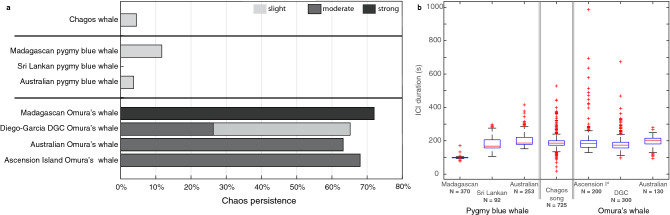
(**a**) Proportion of deterministic chaos (*i.e.*, chaos persistence) in the Chagos song compared with the three Indian Ocean pygmy blue whale song types (Madagascan, Sri Lankan and Australian) and the four Omura’s whale song types (Madagascan, Diego-Garcia DGC, Australian and Ascension Island). Chaos persistence is defined as the proportion of deterministic chaos across the entire song duration (given as a percentage). Shades of grey indicate the strength of the chaos: slight (light grey), moderate (medium grey) and strong (dark grey)). (**b**) Boxplot representation of the Inter-call Intervals (ICI expressed in s) for the different song-types measured in this study. On each box, the central mark is the median, the edges of the box are the 25th and 75th percentiles, the whiskers extend to the most extreme data points considered to be not outliers, and the outliers are plotted individually.

It was difficult to characterize the presence of deterministic chaos where the song (sub)unit was short and the pulse rate was low, as it is difficult to ascertain if the noisy structure (*i.e.*, lack of structure) was part of the whale’s song (*i.e.*, deterministic chaos) or whether it was due to an artefact, such as a sound propagation issue. This was the condition for the subunit 2 of unit 1 of the Chagos song. If this subunit had indeed a chaotic structure, this chaos was slight, and represented 4.5% of the entire duration of the song (Fig. 7a).

Pygmy blue whale songs had only slight deterministic chaos, and of the entire song, it represented: 11.7% of the duration of the Madagascan song; 3.7% of the Australian song; and it was not present in the Sri Lankan pygmy blue whale song (Fig. 7a). In the Madagascan pygmy blue whale songs, slight deterministic chaos was in subunits 1 of both units 1 and 2, and in the Australian pygmy blue whale songs, deterministic chaos was present in subunit 1 of unit 2.

In contrast, deterministic chaos was a significant proportion of all Omura’s whale songs (Figs. 6a–d and 7a). For the song of the Ascension Island Omura’s whale, moderate deterministic chaos was present across 68% of the duration of their song. For the Australian Omura’s whales, deterministic chaos was present across 63.2% of their song, it was moderate-to-strong in the first unit and slight in the second unit. The Madagascan Omura’s whales had strong deterministic chaos across 72% of their song, which excludes the tonal unit as the tonal part was not always present. The Diego Garcia DGC Omura’s whale song had a total chaos persistence of 65.2% (Fig. 7a), with a moderate deterministic chaos present in the first 2.7 s of the song, which represents 26.3% of the song duration (Fig. 7a medium grey section). The song then evolved to a more clearly pulsed sound, with a slightly noisy structure, classified as slight deterministic chaos. Here again, it was difficult to ascertain whether this lack of structure was a characteristic of the song or an artefact of the propagation. Yet, the slight lack of structure was consistently observed across the sampled songs.

#### Inter-call-intervals

Whilst the Madagascan pygmy blue whale had a shorter ICI, all the other acoustic groups studied here had a similar ICI duration (Fig. 7b). Thus, ICI is not a key parameter in the distinction among species and cannot be used to determine whether Chagos-whales are a blue or an Omura’s whale.

### Geographic distribution

Chagos song was detected at 5 of our 6 recording sites at disparate locations across the Indian Ocean, from: the northern Indian Ocean, off Sri Lanka; on both sides of the central Indian Ocean, off the Chagos Archipelago; and in the far eastern Indian Ocean, off northern Western Australia (Fig. 2). The Chagos song was recorded off Sri Lanka (*i.e.*, Trincomalee) in April. Blue whales were observed at the time the recordings were made, and the songs of the Sri Lankan pygmy blue whale were also recorded at the time. The acoustic recording had become degraded as they were made nearly forty years before, on 19 April 1984, and only six distinct Chagos songs were found. Unfortunately, these recordings were of poor SNR which prevented detailed acoustic measurement. The songs, however, had the distinct structure of the Chagos song (Fig. 3) and an ICI of $\simeq$ 200 s (range 200 to 209 s), consistent with the ICI rate measured for the Chagos song off the Chagos Archipelago (Fig. 7b). Further south in the northern Indian Ocean, 6,984 Chagos songs were detected in 2013 (from January to early December) at our recording site RAMA, but no songs were detected at this site in 2012, although recording had been made over a shorter period, from May to December, in that year. In the central Indian Ocean, a total of 486,316 Chagos songs were detected from January 2002 to March 2014 at DGN, and 737,089 Chagos songs from January 2002 to August 2018 at DGS. In the far eastern Indian Ocean, off Kimberley, northern Western Australia, low SNR Chagos songs were manually detected from January to May, in 41 out of the 331 recording days in 2012–2013. In the south-central Indian Ocean, at our recording site RTJ, no Chagos songs were detected in 2018.

**Figure 8:**
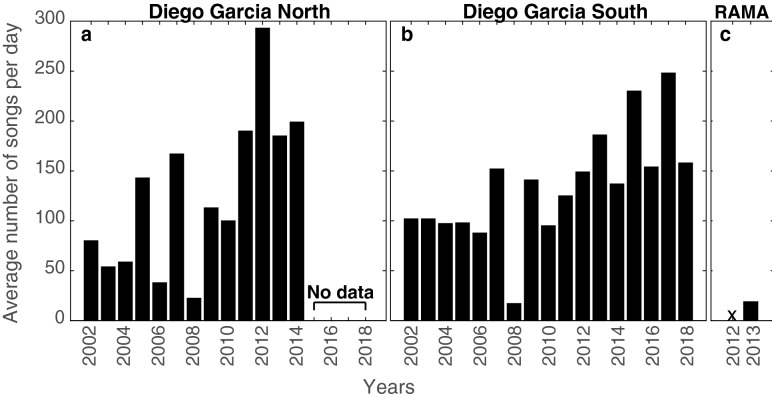
Average number of Chagos songs per day detected in each year of data on the (**a**) western (DGN) and (**b**) eastern (DGS) sides of the Chagos Archipelago, and (**c**) further north-east, at RAMA site.

Figure 8 shows the average number of Chagos songs detected per day for each year of data at the sites located on: (a) the western (DGN); and (b) eastern (DGS) sides of the Chagos Archipelago; as well as (c) further north-east, at RAMA site. The number of songs varied over the years; fewer songs were recorded at both DGN and DGS sites in 2008. In comparison with the Chagos Archipelago sites, the number of songs detected at north-eastern RAMA was low in 2013, with an average of only 20 songs/day.

### Seasonality

**Figure 9:**
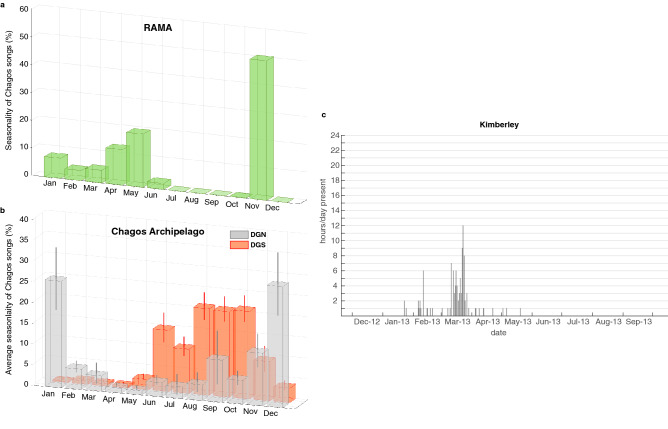
(**a**) Seasonality of Chagos songs at RAMA in 2013, presented as a percentage of songs per month (*i.e.* monthly number of songs divided by total number of songs detected in the year); (**b**) Seasonality of Chagos song averaged over the years (±SE) on the western (DGN—gray) and eastern (DGS—orange) sides of the Chagos Archipelago. This average seasonality is calculated as such: the monthly number of songs is divided by the total number of songs detected in the corresponding year, and averaged over the years. Note that due to the low number of recording days at DGN in 2007 and 2014, and in 2007 at DGS, these years were removed from the averaging (DGN: 11 years and DGS: 15.5 years); (**c**) Hourly presence of Chagos songs in Kimberley (Western Australia) in 2012–2013. Note that the metric and thus the graphic representation used for this site is different from that for RAMA and DGN/DGS: in the Kimberley data set, Chagos songs were logged upon visual inspection of the spectrograms, and a metric of hourly presence/absence of the song per day was used (see the Methods section for details).

Figure 9b shows the average seasonality of Chagos song occurrence on both sides of the Chagos Archipelago. On the western side of the central Indian Ocean (DGN site), Chagos songs were heard predominantly from September to January, with detections peaking in December and January. On the eastern side of the central Indian Ocean (DGS site), songs were detected from June to November, with detection peaks in August to October, depending on the year. In 2013, at the RAMA site (further north-east of the Chagos Archipelago), Chagos songs were detected from January to June (with peaks in May), and in November (Fig. 9a). Off Kimberley, in the north of Western Australia, low SNR Chagos songs were found from the 22 January 2012 to the 20 May 2012, with a peak in March (Fig. 9c).

**Figure 10:**
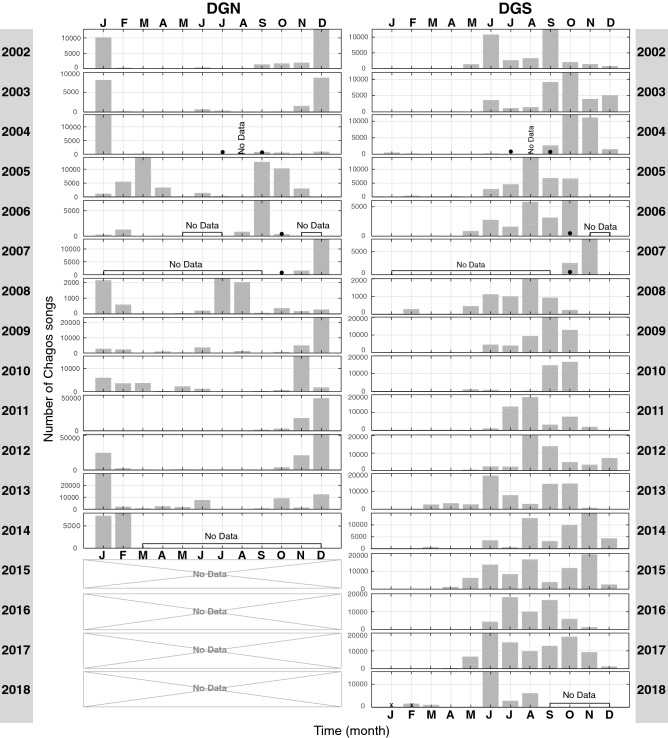
Number of Chagos songs per month for each year at DGN and DGS. Note that the scale of the y-axis differs among years to highlight the seasonal patterns. Months without data are indicated by ‘No Data’, and months with more than 50% of missing days are indicated by a black dot.

We found strong evidence at both Chagos Archipelago sites (DGN and DGS) that the number of Chagos songs changes not only across months (Table 1; $p=0.02417$, Table 2; $p < 0.001$) and years (Table 2; $p < 0.001$, Table 2; $p < 0.001$), but also that there is an interaction between months and years (Table 1; $p < 0.001$, Table 2; $p < 0.001$; Fig. 10). This provides evidence to suggest that there is variation in the pattern of whale songs across years at both sites. Although Chagos songs were detected throughout the year, there were more songs detected at restricted times (Fig. 10). The timing of peaks in song detection was different between the sites. At DGN most songs were detected in 2 to 3 months, whereas at DGS songs were detected over a longer period, from 2 to 6 months. At DGN, where the Chagos song distribution in most years shows clear peaks towards December and January, in a few years, peaks were outside this time (*e.g.*, 2005 in March and September, 2006 in September and 2008 in July and August; Fig. 10). Conversely, in DGS most songs were observed between June and November, although there were inter-annual differences (Fig. 10).

**Table 1:** Assessing the likelihood of an effect on the number of Chagos songs per day at site DGN (n = 3917 days).

Predictor variable	Df	Test statistic	Adjusted *p*-value
Month	11	21.79	0.02459
Year	12	437.12	< 2e−16
Year:Month	110*	1963.07	< 2e−16

Test statistics and *p*-values obtained via type 1 ANOVA likelihood ratio tests from a negative binomial, generalized additive model. *p*-values have also been adjusted to account for multiple hypothesis testing using the Holm adjustment^45^.

*Degrees of freedom are smaller than expected due to some years (n = 4) having missing months.

**Table 2:** Assessing the likelihood of an effect on the number of Chagos songs per day at site DGS (n = 5557 days).

Predictor variable	Df	Test statistic	Adjusted *p*-value
Month	11	197.8	< 2e−16
Year	12	1077.8	< 2e−16
Year:Month	161*	4208.6	< 2e−16

Test statistics and *p*-values obtained via type 1 ANOVA likelihood ratio tests from a negative binomial, generalized additive model. *p*-values have also been adjusted to account for multiple hypothesis testing using the Holm adjustment^45^.

*Degrees of freedom are smaller than expected due to some years (n = 4) having missing months.

## Discussion

**Figure 11:**
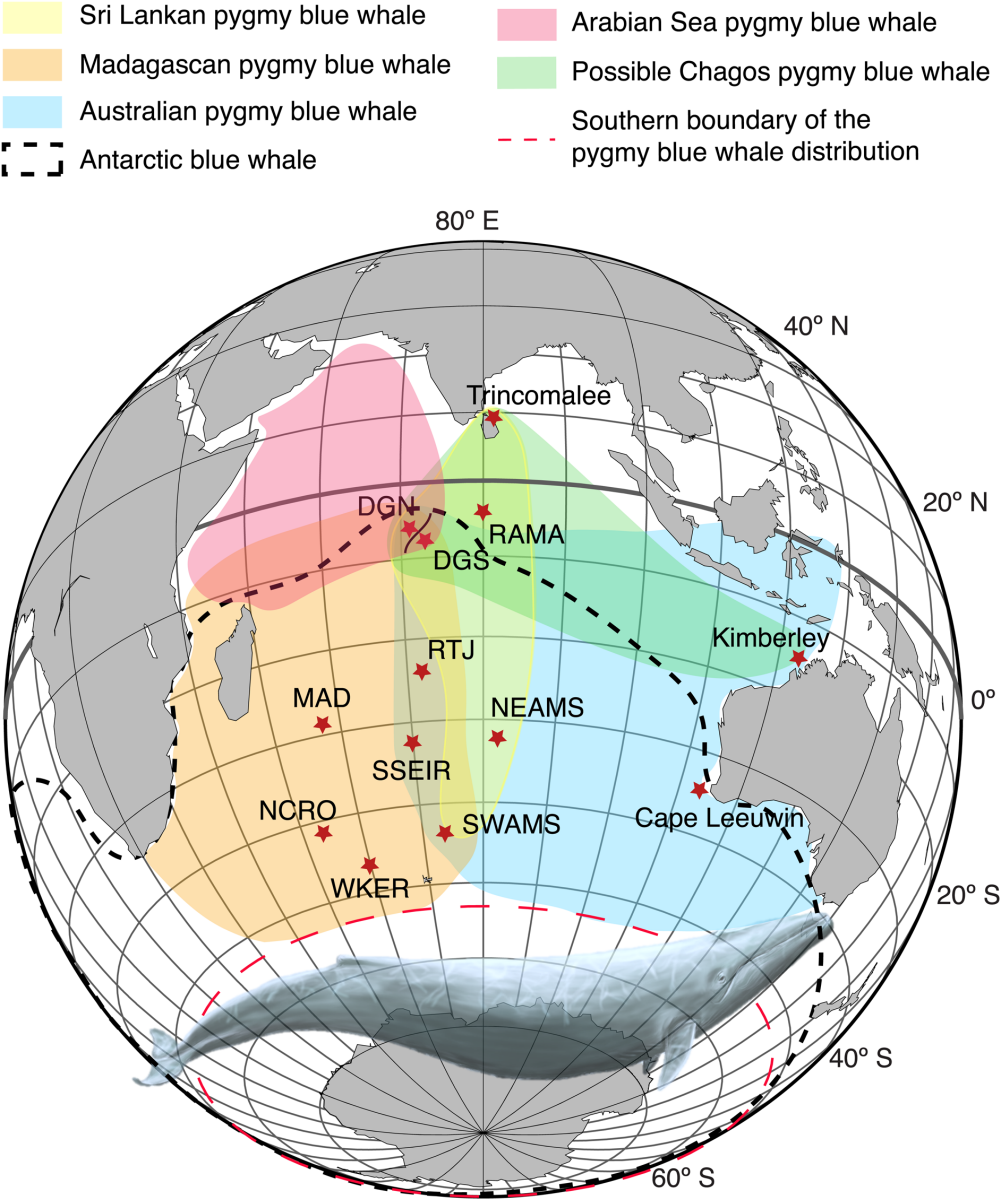
Distribution of the five blue whale acoustic populations of the Indian Ocean: the Sri Lankan—NIO (yellow); Madagascan—SWIO (orange); Australian—SEIO (blue); and Arabian Sea—NWIO (red) pygmy blue whales; the hypothesised Chagos pygmy blue whale (green); and the Antarctic blue whale (black dashed line). These distributions have been inferred from the acoustic recordings conducted in the area (*e.g.*,^7–9,11,13,21,50,51^, and E.C.L personal observations). The long-term recording sites used to infer these distribution areas are indicated by red stars. The map was generated with GMT^52^. Blue whale illustration by Alicia Guerrero.

We suggest that there is a previously unknown pygmy blue whale acoustic population, the Chagos blue whale, in the central Indian Ocean. These whales migrate between the waters of the central and northeastern Indian Ocean. The songs of the Chagos blue whale represent a large part of the soundscape in the equatorial Indian Ocean and have done so for nearly two decades. A high number of songs detected across 17 years of continuous acoustic data suggests that they were produced by a large number of whales, rather than by a few individuals. Until very recently, it was believed that there was only one pygmy blue whale population, the Sri Lankan pygmy blue whale, in the northern Indian Ocean^3,7,46–49^. In 2020, Cerchio et al. showed the existence of another pygmy blue whale population in the north-western Indian Ocean. We now suggest that there is a third acoustic population, located in the central and north-eastern Indian Ocean (Fig. 11).

Our findings support Sousa and Harris (2015) proposal that the Chagos song, which they referred to as ‘DGD’ call, is produced by a blue whale. Although we propose that rather than ‘Diego Garcia Downsweep’, or DGD call, it is renamed as the Chagos song because it is a three-unit complex sound, repeated in sequence, rather than a true ‘downsweep.’ The comparison of the Chagos song with the Indian Ocean pygmy blue whale songs and the Omura’s whale songs indicates that the Chagos song is likely produced by a pygmy blue whale. Although Sousa and Harris (2015) recorded the Diego Garcia Omura’s whale (DGC) song alongside the recordings of the Chagos (DGD) song^14^, we found no support for the hypothesis that the Chagos song was also produced by an Omura’s whale.

We show that the pygmy blue whale songs have a complex structure, with multiple unit types different in nature including: simple tones to harmonic, pulsed and biphonation sounds. The structure of the pygmy blue whale song is indeed more complex than previously described^7,8,53^, and perhaps this complexity is linked to their vocal apparatus^54–56^. Note that such thorough description of the (blue) whale song structure is rarely undertaken and, put in parallel with the recent knowledge about mysticete vocal apparatus^54–56^, may help understanding how their songs are produced. The Chagos song shares with the pygmy blue whales the acoustic complexity, especially in the first unit which starts pulsed, continues as a pulsed sound with a frequency upsweep, and then becomes a tonal frequency-modulated sound. Interestingly, the first unit of the Chagos song is so similar to the first unit of the Sri Lankan pygmy blue whale song (*i.e.*, both are non-tonal pulsed sounds with a similar pulsed rate of $\sim$ 3.2 Hz), that it is easy to misidentify the first unit of Chagos song with the first unit of the Sri Lankan song when the recordings are of poor SNR. If, as suspected by Mc Donald et al., blue whales show geographic similarities in their songs^5^, this might explain similarity between the first units of the Chagos and the Sri Lankan blue whale songs and argues in favour of a blue whale source species for the Chagos song. Also, similarly to blue whale songs, deterministic chaos was present in a small proportion of the Chagos song, and was only slight (Fig. 7).

By contrast, Omura’s whale song-types have very different features to Indian Ocean pygmy blue whales, which seem to be highly characteristic of the Omura’s whale species (Fig. 6) and independent of their geographic origin: their energy is distributed between 15 and 55 Hz, with a peak around 20 Hz and another one around 40–45 Hz. The pulse rate of their pulsed units is very low (< 2.3 Hz) and unlike the blue whale songs they are all characterized by a high proportion of a moderate to strong deterministic chaos (> 60%). Finally, their songs are likely to contain biphonation and even triphonation sounds with two or three very clear independent fundamental frequencies. None of these very particular characteristics are shared by the Chagos song.

We also show that the Chagos song has been produced with a gradual frequency decline over time, for the past two decades (Fig. 4). This progressive decline in frequency (a downwards shift) is a trait observed worldwide for blue whale songs^36,38,39,57,58^. We argue that the frequency decline observed in the Chagos song is further support in favour of the Chagos song being produced by a blue whale.

Allometry studies have shown a relationship between the size of an animal and their acoustic behaviour, for example, larger mammals produce lower frequency sounds than smaller mammals, as they have a larger vocal apparatus^59,60^. Although an allometric relationship has yet to be established between the source level of the sound a mammal produces and its body size, it is likely that larger mammals, with larger vocal apparatus, are capable of making louder sounds than smaller mammals. The Chagos song is loud (*i.e.*, 187 ± 6 dB re:1 $\upmu \hbox {Pa}$ at 1 m in the frequency range of 15-60 Hz^61^), well within the source level estimates reported for blue whale songs (*i.e.*, range from 174 to 196 dB re:1 $\upmu \hbox {Pa}$ at 1 m^6,62–65^). Although the source level of the Omura’s whale song is unknown, the smaller body size of the Omura’s whale (*i.e.*, 12 m^66^), similar to the Bryde’s, minke and sei whales, would suggest that they produce songs of similar source level (*i.e.*, range from 147 to 169 dB re:1 $\upmu \hbox {Pa}$ at 1m^67–69^). These are relatively wide source level ranges, that incorporate uncertainties as well as the inter-individual variations, however there is no overlap between the source level ranges reported for blue whales with the smaller baleen whales. Thus, it seems safe to postulate that the high source level of the Chagos song, well within the blue whale range, is further indication that it is produced by a large baleen whale, the size of a blue whale, rather than the much smaller Omura’s whale.

Note that Sousa and Harris ruled out the possibility of a Bryde’s whale source species of the Chagos song, based on the time and frequency characteristics of the sounds^14^. As the vocal repertoire of the Bryde’s whale is still not well known, one might argue that the Chagos song is part of it. However, despite the scarcity of reports of Bryde’s whale vocalisations along with sightings, the described sounds are mostly pulses and “bursts” (often used to describe sounds with deterministic chaos), and are characterised by a short duration (< 3 s), a frequency ranging from 76 to 208 Hz, and a source level of 155 dB re:1 $\upmu \hbox {Pa}$ at 1 m (*e.g.*^69–72^). There is nothing alike in the Chagos song. Moreover, prior its description as a new species, the Omura’s whale was mistaken for a Bryde’s whale^66^, and Omura’s whale vocalisations have been first described in 2015^15^. It is thus possible that some of the sounds described as possible Bryde’s whale vocalisations were in fact Omura’s vocalisations. We suspect that it is the case for the sound recorded off New Zealand by McDonald^73^, which on the spectrogram (see Fig. 4D in^74^) resembles to an Omura’s whale song. Based on the great dissimilarity in duration, frequency and source level between the ascertained Bryde’s whale vocalisations and the Chagos song, it is safe to assume that the latest is not emitted by a Bryde’s whale.

The Chagos songs are detected at disparate locations; from the central Indian Ocean, around the Chagos Archipelago, moving further east to the north of Western Australia, and likely further north to Sri Lanka, off Trincomalee, as suggested by the recording from 1984. The Chagos songs found off north of Western Australia were all of low SNR. This suggests that the whales producing these songs were at the limit of the detection range. Estimating the detection range of the Chagos song is beyond the purpose of this study, but as it has a similar source level than blue whale songs (see above), it has an equivalent detection range. In general, the detection range of blue whale songs has been reported from up to 200 km for the Antarctic blue whale^65,75^ to 300 km for the pygmy blue whale^75^. In the same area, off north of Western Australia, Mc Pherson et al. calculated a detection range of 80 km for the Australian pygmy blue whale^76^. Many variables will change these estimates, for example, environmental conditions surrounding the hydrophone, the noise platform level, the source level of the songs, the depth of the vocalizing whale, as well as the bathymetry of the region^61,77^. Therefore, the Kimberley site is at the limit of the Chagos whale distribution area, and the songs detected in the recordings have likely been produced either few hundred of km further offshore, or possibly off south Indonesia.

These Chagos whales show thus the wide-ranging distribution of other blue whale populations. To date, the Chagos songs have not been detected in the western Indian Ocean, either in the southwest, around Reunion Island^78^ and in the Mozambique Channel, or in the northwest, off Oman (S. Cerchio, pers. comm.), nor have they been detected in the southern Indian Ocean (south of 26$^{\circ }$ S) (^11,21,79^ and E.C.L. personal observations). This indicates that the Chagos whales migrate between the central tropical waters, the eastern tropical to subtropical waters, and possibly the equatorial northeastern waters of the Indian Ocean. They do not undergo a migration towards the western Indian Ocean off the African coast, nor to the Southern Ocean. The Maldives, in the tropical north Indian Ocean, is a location where there have been numerous blue whale sightings, strandings and catches^48,49^. However, as yet, the acoustic identity of those whales remains unknown. It is possible that these are Chagos blue whales, or that these Chagos whales visit this region alongside other blue whale acoustic populations, such as the Sri Lankan pygmy blue whale.

Little is known about Omura’s whale behaviour. Yet, they have been detected year-round off north of Western Australia, showing very limited migration mouvements, that appear to happen along the coast^17,76,80^. Similarly, the Madagascan population is described as a resident population, with a distribution limited to the central west and northwest coasts of Madagascar. Both these Omura’s whale acoustic populations have been recorded year-round, with no clear and regular seasonal patterns^43,76,80^.

On the contrary, very clear and repeated seasonal patterns have been observed for the Chagos songs. In the central tropical Indian Ocean, the number of Chagos songs oscillates seasonally between one side of the Chagos Archipelago and the other, and do so across the 17 years studied. Where the Chagos whales are present off the west side of the archipelago from September to January, their songs are detected off the east side from June to November. Our results are consistent with the daily presence/absence of their songs in 2002-2003 as reported by Sousa and Harris^14^. This seasonal oscillation in song detections indicates that the whales migrate annually from the western across to the eastern tropical Indian Ocean. This migration pattern may be driven by environmental conditions such as shifts in the Northern Indian Ocean. The Northern Indian Ocean is influenced by the complex monsoonal system in the area which drives upwelling and productivity, and thus, it is likely influential in the distribution of the blue whale populations. Anderson et al. highlighted a possible food supply at Diego Garcia from July to August onward^49^. This corresponds with the time we detect Chagos whales to the east of the archipelago (*i.e.*, at DGS).

The two Chagos Archipelago recording sites (DGN and DGS) are in close proximity (*i.e.*, 220 km), however they are believed to be independent acoustic sampling areas. Thus, the same whale song is not recorded simultaneously at these sites. The reason why these two hydroacoustic recording sites were set up by the Comprehensive Nuclear Test-Ban Treaty Organisation is because the Chagos Bank, which supports its archipelago, with its shallow depth and long north-south extension, acts as an acoustic barrier between the western and eastern equatorial Indian Ocean. Sounds produced on either side of the Chagos Bank are unlikely to be heard on the other side^77^, except perhaps for low frequency sounds below 30 Hz^81^. Since most of the energy of the Chagos song lies in frequencies above 30 Hz, especially the component we used in our detection process (*i.e.*, the first unit of the song) it is safe to consider that, for these songs, the northern site (*i.e.*, DGN records) represents the soundscape west of the island, whilst the southern site (*i.e.*, DGS records) the soundscape east of the island. In addition, the difference in the seasonal occurrence of Chagos songs between these western and eastern sites (*i.e.*, DGN and DGS) indicates that, despite the rather small distance between the recording locations, they are likely independent sampling areas. A direct comparison of the abundance of songs between our Chagos Archipelago sites would be hazardous given that the sound propagation conditions in the area are not well understood^77^ and the detection range may differ between the two sites.

The seasonal pattern of the Chagos song presence off Kimberley corresponds perfectly with the period when they are absent from the Diego Garcia recordings: austral autumn. This confirms the previous hypothesis of a migration from the west of the Chagos Archipelago to the eastern tropical Indian Ocean. This also correlates with the observations made the same year (2013) at the RAMA site, located further north-east of the Chagos Archipelago. At RAMA, songs were detected from January to June, with a peak in April-May, which seems to confirm that after leaving the Chagos Archipelago at the end of austral summer (around February), the Chagos whales moves eastward, and might spread up to Indonesia and north of Western Australia. The absence of songs in 2012 may be due to our recordings starting in May, potentially after the time the Chagos whales have migrated. Indeed, the seasonal patterns for the Chagos Archipelago (*i.e.*, at DGN and DGS) show that despite overall stability, there is inter-annual variability, particularly in the date of arrival of the whale songs. Alternatively, it is possible that the attendance at this site is much more variable and does not occur every year. Indeed, as RAMA is located in deeper waters (see Fig. 2), away from any seamounts or islands, it is likely to be a less productive region than other areas. The peak of detection in November 2013 is quite puzzling here. These detections spread throughout the month, so they are not the fact of one single individual roaming around the hydrophone. Yet the site is still relatively closed to the DGS site, where Chagos songs are detected during austral spring, which could explain their presence at RAMA in November. The interpretation of the seasonal presence of Chagos songs at RAMA remains quite limited given the short duration of our recordings (*i.e.*, 16 months) relative to our Chagos Archipelago sites. Nevertheless, the detection of Chagos songs at RAMA and north of Western Australia from January to June 2013 indicates that they are emitted year-round and not only seasonally.

Finally, the Chagos song was present in April in the equatorial waters of the northeastern Indian Ocean, off Sri Lanka. Unfortunately, the data recorded off Trincomalee, Sri Lanka^46^ were too degraded to make any further inference, and cannot be more than an indication of a punctual presence of the Chagos whale around east Sri Lanka in autumn. Yet, this timing of presence fits with the above hypothesis of a clockwise migration, moving from east to west in the central tropical Indian Ocean (around Chagos Archipelago) between June and January, and then spreading further east and north-east, as far as Sri Lanka, from the end of January to the end of May. Such east-to-west migration does not fit with the “usual” north-south migration patterns between feeding and wintering grounds generally observed for blue whales. However, as underlined above, the Indian Ocean is a complex system and its productivity is linked to the monsoons, with a ’winter monsoon’ from October to April with winds blowing from the North-East, and a ’summer monsoon’ from April to September with south-westerly winds. This east-to-west oscillation may be the reason for such east-to-west whale migration. Further acoustic recordings are required to ascertain the acoustic identity of the blue whales regularly seen in the northern Indian Ocean waters, particularly off Sri Lanka^82–84^, off Indonesia as well as off the Maldives.

Acoustic structure of the songs, geographic repartition and seasonality argue thus in favour of the Chagos song being produced by a blue whale. Moreover, it might be safe to go further in saying that the Chagos song is likely produced by a pygmy blue whale, given the known geographic distribution of pygmy versus Antarctic blue whales, estimated using the size of the whales caught during the industrial whaling and reported in the whaling data^85^. The Chagos song may not only be a new blue whale song-type, but the seasonal and geographic pattern of detections across the Indian Ocean is evidence that these whales are a separate acoustic group migrating between the central and eastern tropical and equatorial Indian Ocean. Indeed, the high number of Chagos songs recorded around Diego Garcia suggests that multiple Chagos whales are present. For instance, as the Chagos songs have an average ICI of 191 s, one Chagos whale, singing continuously, will produce 18 songs in an hour. Thus, the hourly rate of 110 songs/hour recorded at DGS on 8 November 2004 suggests that at least six Chagos whales were singing on that day, within range of the hydrophone.

The seasonal and geographic patterns of the Chagos acoustic population are clearly different from the patterns for all other pygmy blue whale populations (Fig. 11). Thus, we can be confident that the Chagos song is not a variant song of an existing pygmy blue whale population. At Diego Garcia in 2002–2003, Sri Lankan pygmy blue whale songs were detected mostly from March to June at DGN, and in May–July and October–February at DGS^7^. These patterns are different to those of the Chagos song. In addition, the observations made on the data during the double-check of the detections showed that Sri Lankan songs were much more numerous at DGN than at DGS (E.C.L. personal observations). Sri Lankan songs have been recorded year-round further south, at $\sim$ 31$^{\circ }$ S, 83$^{\circ }$ E, and down to 42$^{\circ }$ S, 74$^{\circ }$ E, with a peak in austral summer and a secondary peak in winter^8,11^. No Chagos songs have been recorded at these locations (^21,58,79,79^, and E.C.L. personal observations).

The Madagascan pygmy blue whale songs have been recorded on very limited occurrence and only on the west side of the Chagos Archipelago (DGN) in May–July 2002–2003^7^, which is when Chagos songs are absent from the area. The Madagascan pygmy blue whale are known to dwell in the west and central Indian Ocean, and have been recorded at up to 46$^{\circ }$ S and 74$^{\circ }$ E^11^.

Finally, the Australian pygmy blue whales, although present at the Kimberley site, they are detected rarely in Chagos Archipelago waters (^7^ and E.C.L personal observations).

## Conclusion

We suggest that the Chagos song is produced by a population of pygmy blue whales that migrates between the central Indian Ocean and eastward to north Western Australia and Indonesia, and likely as far north as the equatorial Indian Ocean, off Sri Lanka (Fig. 11). The diversity of pygmy blue whale acoustic populations in the northern Indian Ocean is greater than expected, with at least three acoustic populations in the central and northern Indian Ocean (including the newly discovered acoustic population in the northwestern Indian Ocean^13^), rather than a single resident population, as previously believed^49^. Further effort is needed to better understand the distribution of blue whale populations in the northern Indian Ocean, and especially to ascertain the acoustic identity of the blue whales regularly observed off Sri Lanka, as simultaneous acoustic and visual observation are needed to confirm the species producing the Chagos song. This possible new discrete acoustic population of blue whales should be taken into consideration in future conservation efforts^86^.

## Methods

### Data acquisition

**CTBTO sites** The hydroacoustic data sets from the central Indian Ocean were obtained from the International Data Centre of the Comprehensive Nuclear Test-Ban Treaty Organisation (CTBTO) in Vienna. We used the data recorded at the CTBTO hydrophone station HA08, located off Diego Garcia Island, an atoll of the Chagos Archipelago, in the central Indian Ocean, south of the Equator. This station is comprised of two hydrophone triplets: the northern one, H08N, referred as Diego Garcia North (DGN, 06.3$^{\circ }$ S, 071.0$^{\circ }$ E) and consisting of the hydrophones H08N1, H08N2, H08N3, and the southern one, H08S, referred as Diego Garcia South (DGS, 07.6$^{\circ }$ S, 072.5$^{\circ }$ E) and consisting of the hydrophones H08S1, H08S2, H08S3. DGN and DGS are about 220 km apart. The data were acquired between 1 January 2002 and 1 March 2014 at DGN, and 1 January 2002 and 14 August 2018 at DGS. These recordings were from autonomous hydrophones moored in the SOFAR channel and cabled to Diego Garcia Island (for details see^14^). The sampling rate was 250 Hz. In this study, we used the recordings from the hydrophones H08N1 at DGN and H08S1 at DGS. The records are almost continuous, with the exception of data gaps and for 2007, where less than 3 months of recordings were available at both sites (see Fig. 10).

We also used the CTBTO data recorded off Crozet Island in 2004 (HA04, hydrophone H04N1, 46.2$^{\circ }$ S, 051.8$^{\circ }$ E), and off Ascension Island in 2009 (HA10, hydrophone H10N1, 7.8$^{\circ }$ S, 14.5$^{\circ }$ W). The Madagascan pygmy blue and Omura’s whale songs were extracted from these recordings respectively.

**IMOS sites** The hydroacoustic datasets from the eastern Indian Ocean were recorded by the Australian Integrated Marine Observer System (IMOS) at the Kimberley site (Western Australia (WA); 15.5$^{\circ }$ S, 121.25$^{\circ }$ E) from 20 November 2012 to 17 October 2013 and at Perth Canyon site (WA; 31.9$^{\circ }$ S, 115.0$^{\circ }$ E) in 2009. They were downloaded via the IMOS online platform (https://acoustic.aodn.org.au/acoustic/). These recordings are from single fixed hydrophones that record ocean sounds for 500 s of every 900 s at a sampling rate of 6,000 Hz (upper frequency limit of 2,800 Hz at -3 dB). Data were subsampled to 250 Hz.

**OHASISBIO sites** Hydroacoustic recordings from the OHASISBIO hydrophone network^87^ were used from two sites: 16 months from RAMA (03.8$^{\circ }$ S, 080.5$^{\circ }$ E) from 4 May 2012 to 10 December 2013 and 12 months from RTJ (24.0$^{\circ }$ S, 072.0$^{\circ }$ E) in 2018. These recordings were from autonomous hydrophones moored in the SOFAR channel, at a sample rate of 240 Hz (see^21,87,88^ for more details).

**Sri Lankan site** The acoustic data were collected in 1984-1985 off Trincomalee, Sri Lanka using a hydrophone directly from a boat (for details see^46^). However, most of these data are degraded, and only few segments, recorded on 23 March1984 (duration $\sim$ 45 min), 19 April 1984 (duration $\sim$ 16 min), and 8 May 1985 (duration $\sim$ 16 min), were still usable. The data, originally sampled at 96 kHz, were down-sampled at 250 Hz to scrutinise the low-frequency signals.

### Song measurements

High quality songs were extracted from the data set to measure their spectral and temporal features. The selected songs were carefully chosen upon visual inspection to make sure that their SNR was high enough to allow the most precise measurements possible. The measurements were performed on spectrogram and PSD representations for each song, using a customised Matlab code (spectrogram parameters: Hann window, 90% overlap, 1024-point FFT). The Matlab code PAMguide^89^ was used to plot the relative PSD of the Omura’s whale songs (see Fig. 6 lower panels).

The interval between two successive songs (ICI, Inter-Call Interval) was also measured. It is defined as the interval between the beginning of one song and the beginning of the following song. The ICI was measured in the song sequences within which the individual songs used for the feature measurements were selected. Because measuring ICIs does not require very high SNR, the ICIs were measured provided that the song start and finish was clearly visible and provided that, in case of the presence of multiple singers, it was possible to differentiate the song sequences of different animals (*i.e.*, one in the near field, the other in the far field). ICIs were measured using Raven Pro V1.6^90^. As most of the measured ICIs were extracted from the same song sequence, resulting in auto-correlation, we could not perform statistics on ICIs measurement.

The number of analysed songs, their provenance, and recording dates are presented in Table 3.

**Table 3:** Recording sites and dates, and number of analysed Chagos, pygmy blue and Omura’s whale song-types.

Song type	Recording site	Hydrophone network	Number of analysed songs	Year	Days
Chagos songs	DGS - H08S1	CTBTO	146	2017	6-10/06, 11/06, 13-15/06, 30/06;
					19/07, 21-22/07, 24-25/07, 29/07;
					4-5/08, 17/08, 21-22/08, 24/08, 30/08;
					2/09, 4-5/09, 22/09;
					2-3/10, 23/10, 26/10;
					2/11
Madagascan pygmy blue whale	Crozet - H04S1	CTBTO	92	2004	9/03;
					10/04, 14-15/04, 23/04, 25/04
Sri Lankan pygmy blue whale	DGN - H08N1	CTBTO	74	2009	14/02;
					19/04;
					4/05
Australian pygmy blue whale	Perth Canyon (WA)	IMOS	112	2009	28/02;
					6/03, 17/03, 21/03, 23/03, 25-26/03, 28-29/03, 31/03;
					1/04, 3-4/04, 7/04, 29-30/04;
					1/05, 6/05, 10/05,18/05, 22/05
Ascension Island Omura’s whale	Ascension Island - H10N1	CTBTO	60	2005	5-6/11, 10-11/11;
					20/12
Diego Garcia DGC Omura’s whale	DGN - H08N1	CTBTO	80	2003	23-24/01, 27/01;
					4/02
Australian Omura’s whale	Kimberley (WA)	IMOS	100	2012	22/09;
					28/11;
					20/12
				2013	2-3/01, 10/01;
					3-6/03, 15/03;
					7/04, 30/04

### Song detection

To detect individual Chagos songs in a long-term data set, we used an automated detection algorithm. This algorithm performs a dictionary-based detection by modelling mysticete calls with sparse representations^91^. This method uses a decision statistic that offers optimal properties with respect to false alarm and detection probabilities^92^. The signal of interest is thus modelled using a dictionary, which allows the use of this detector for previously unknown or understudied recurrent signals. In addition, this method does not suffer from the drawbacks of detection methods such as spectrogram correlation (see^91,92^ for further details). To overcome the effect of any potential variation of the call characteristics across the years on the detector performance^36,37,57,58^, we created a dictionary for each year of data, using signals with different signal-to-noise ratios, manually picked up at different periods of the year, during a first visual exploration of the data set. The detection threshold, which can be interpreted as an estimate of the signal-to-interference-plus-noise ratio, and which measures the match between the observed data and the assumed sparse representation of the call to detect, was set to -8.6 dB after a prior analysis performed on a manually annotated dataset composed of 1550 Chagos songs. These tests resulted in a recall level of 0.93 and a precision of 0.92, although we acknowledge the difficulty to use a manually annotated dataset as ‘the ground truth’ for assessing detector performances^58,93^. All the detections were checked and the false detections were manually removed. We obtained an average of 0.33 ± 0.08 (mean ± S.E.) false alarm/hour for the DGN data, 0.06 ± 0.012 false alarm/hour at DGS and 0.10 false alarm/hour at RAMA. The higher false alarm rate at DGN is mainly due to a greater presence of other whale calls, especially from Omura’s whales and Sri Lankan pygmy blue whales, which can be detected as Diego Garcia songs.

The IMOS acoustic data recorded off Kimberley contained very low SNR Chagos songs, often barely visible on the spectrograms. Given the format of the recordings (5 min every 15 min), as well as the very low SNR that would probably have prevented any efficient automated detection, Chagos songs were logged using Raven Pro upon visual inspection of the spectrograms, and a metric of hourly presence/absence of the song per day was used.

### Statistical analysis

In order to assess the consistency of whale call distribution in months across years, the number of calls per day at the two Chagos’ sites, DGN (n = 3917 days) and DGS (n = 5557 days), was analysed separately with a negative binomial, generalized additive model using the mgcv package in R^94,95^. Fixed effects for ‘year’ and ‘month’ were included as the predictors of interest against the response of ‘calls per day’. These fixed effects and their interactions were specified in order to assess seasonal and yearly changes to the mean abundance of calls . A negative binomial family was utilized to account for a strong mean-variance relationship of the count data that was over-dispersed relative to the Poisson distribution. A generalized additive model was then chosen to employ a cyclic smooth term for day of the year to account for daily temporal autocorrelation and an offset term was also applied to account for total monitoring hours per day. P-values have also been adjusted to account for multiple hypothesis testing using the Holm adjustment^45^.

## Data Availability

The number of detected songs per month at each site are available at [to be updated].
